# Sex-specific associations between systemic autoantibodies and allergic sensitization or allergic disease – results from a population-based study

**DOI:** 10.3389/fimmu.2025.1740193

**Published:** 2026-01-16

**Authors:** J. Linseisen, C. Laichinger, E. Kling, R. Hoffmann, F. Rohm, C. Meisinger

**Affiliations:** 1 Epidemiology, Medical Faculty, University of Augsburg, Augsburg, Germany; 2 Institute for Laboratory Medicine and Microbiology, University Hospital Augsburg, Augsburg, Germany

**Keywords:** allergy, antinuclear antibody (ANA), autoimmune antibody (AAB), autoimmunity, Bavarian food consumption survey II (BVS II), immunoglobulin E (IgE), population-based study, rheumatoid factor (RF)

## Abstract

**Objective:**

So far, knowledge of determinants of the presence of systemic autoantibodies (AABs) in the population is limited. Here, we investigated possible associations between serum AABs and allergies, using data on allergic sensitization and diagnoses of allergic diseases.

**Methods:**

In 331 participants of a population-based study, 5 humoral systemic AABs and 7 AAB screening tests were analyzed. Allergic sensitization was characterized by specific IgE concentrations in serum samples (CAP class ≥ 2); additionally, self-reported diagnoses of allergic diseases were used as exposure variables. Multivariable adjusted logistic regression models were applied to explore the association with AAB test positivity; all analyses were stratified by sex. In a sensitivity analysis, AAB test results were defined as non-normal and normal.

**Results:**

In 46.2% and 37.1% of female and male study participants, respectively, at least one positive AAB test was identified. Allergic sensitization was observed in 23.8% and 29.7% of female and male participants, while 26.2% and 9.3% reported at least one diagnosis of allergic disease, respectively. Positive associations between allergic sensitization and serum AABs were identified in women for rheumatoid factor (RF), antinuclear antibodies (ANA), and at least one positive AAB test; in men, there was some indication for an association with anti-neutrophil cytoplasmic antibody (ANCA) positivity. Self-reported diagnosis of allergic diseases was not significantly associated with the AAB positivity.

**Conclusion:**

In this population-based group of adults, there is evidence for an association between allergic sensitization and systemic AABs, almost exclusively in women. Large prospective studies are needed for confirmation and further investigation of individual AABs.

## 1 Introduction

In clinical practice, the significance of organ-specific autoantibodies (AABs), isolated or as a set of few related AABs, is well established. However, as population-based studies started to estimate AABs, it became clear that the prevalence of systemic AABs is quite substantial. For example, in the NHANES study, a prevalence of 39% in women and 22% in men was reported when analyzing a total of eight AABs (1). This indicates that the presence of serological autoimmune markers does not necessarily indicate clinical autoimmune disease (AID); rather, it does mark the presence of biologic autoimmunity to a much greater extent than previously assumed.

Several systemic AABs occur with higher frequency in individuals suffering from allergic diseases. For example, antinuclear antibodies (ANAs) appear more frequently in allergic individuals compared with non-allergic persons (2, 3). Another study reported associations in celiac disease between AABs and immunoglobulin E (IgE) sensitization (4). Thus, there is some indication of a link between allergies or allergic sensitization and specific AAB profiles, suggesting overlapping allergy–autoimmunity features.

In contrast to many chronic diseases, risk factors for the occurrence of circulating AABs in population-based samples were not well described. For example, smoking and obesity were investigated with mixed results (5). Female gender, however, was consistently linked to higher prevalence of serum antibodies associated, e.g., with rheumatoid arthritis (RA) and thyroid disorders (2, 6), and this was also described for the participants in the present study investigating a broad range of AABs (5). Also, women have a much higher burden of AID as compared to men (7). Immunological alterations during pregnancy and the role of sex hormones, especially estrogens, are discussed as underlying mechanisms (8). Concerning the frequency of allergic sensitization in the general German population, no clear gender difference was observed, while the number of diagnoses of allergic diseases is distinctly higher in women than in men (9, 10).

Here, we explore in an adult general population whether associations exist between several systemic AABs and screening tests and allergic sensitization or allergic diseases. Due to the observed differences in AABs prevalence, all analyses were conducted separately for women and men.

## 2 Methods

### 2.1 Study design and data collection

The data analyzed in this study were collected as part of the Second Bavarian Food Consumption Survey (BVS II), a mono-centric study conducted between 2002 and 2003. This cross-sectional study assessed dietary intake and lifestyle factors among a random sample of 1,050 individuals aged 13 to 80 years from the Bavarian population in Germany. We included free-living individuals in the age range of 13–80 years (18–80 years for blood collection) identified by random sampling and willing to participate in the study and to provide informed consent. Exclusion criteria were language barriers and other reasons for not understanding the study information, thus unable to give informed consent.

Data collection was performed using standardized computer-assisted personal interviews and included information on socio-economic status, lifestyle behaviors, health status, diagnosed diseases, and medication use.

Adult participants (aged ≥18 years) who had completed at least one 24-hour dietary recall and provided information on physical activity (n=879) were invited for blood sampling and further anthropometric assessments. Of these, samples from 568 participants were obtained.

All participants provided written informed consent. The BVS II study was approved by the Ethics Committee of the Bavarian Medical Association (Bayerische Landesärztekammer) on June 19, 2002 (No. 02111). The BVS II study was conducted in accordance with the principles defined in the Declaration of Helsinki.

### 2.2 Blood sampling

Venous blood samples were taken from 568 participants, chilled at 4°C and centrifuged, aliquoted, and stored at -80°C (serum) or -20°C (red blood cells, RBC) until analysis. Pre-processing of the blood samples (centrifugation, aliquoting) was performed in the local health offices, and the samples were transported to the central freezers on the same day according to standard specifications. The influence of pre-analytical variables on sample quality can therefore be regarded as low. Continuous tracking of temperature enabled the safe and efficient storage of samples, which is essential for the long-term stability of biospecimens. Measurements of specific IgE and AABs were conducted in serum, while fatty acid profiles were measured in RBC membranes. The analysis of RBC membrane fatty acids was conducted no later than 6 months after blood drawing. Serum samples used for IgE determination were stored for a maximum of 1.25 years, and AAB analysis was performed about 17 years after sample collection. Due to the requirement to use previously unthawed serum samples, a set of 331 samples was selected for analysis of AABs; this selection is based on sample availability, and availability is very likely at random. 17 years after blood collection (in 568 persons), no full set of unthawed plasma or serum samples was available anymore. The characteristics of the participants in the present study and in the full group (with blood collection) are given in the **Supplementary Table S1**.

After the exclusion of eight individuals with a diagnosis of cancer, data from 323 participants remained for the present analysis.

### 2.3 Allergic sensitization and allergic diseases

Information about allergic sensitization was derived by specific serum IgE measurements using the CAPSX1 *in-vitro* screening test (Pharmacia Upjohn, Uppsala, Sweden). This ELISA-based test, also known as Phadiatop test, detects the presence of specific serum IgE against common allergens (timothy, rye, birch, mugwort pollen, house dust mite (Dermatophagoides pteronyssinus), cat and dog epithelia, as well as Cladosporium herbarum mould). All serum samples were analyzed in a blinded manner according to the manufacturer’s recommendations at the Federal State Health Office Baden-Württemberg, and the results were categorized in seven CAP classes. A result of CAP class 2 or higher, i.e., at least one specific IgE ≥700 U/l, was considered as allergic sensitization. Other researchers also used CAP classes ≥1 to define allergic sensitization. According to Fall and colleagues CAP class 1 represents low IgE concentrations, and CAP class 2 moderately increased IgE concentrations (11). We added additional analysis comparing the results of both definitions.

During the face-to-face interview, study participants were asked about ever diagnosis (by a physician) of allergic diseases, including the IgE-mediated allergies allergic rhinitis, atopic dermatitis/neurodermatitis, and food allergy; we also included asthma, though we could not separate allergic asthma. We defined “at least one diagnosis of allergic disease” as an exposure variable, comprising information on asthma, allergic rhinitis, atopic dermatitis/neurodermatitis, and food allergy.

### 2.4 Definition of positive findings for ABBs

Serum samples were analyzed by indirect immunofluorescence tests (IIFTs), ELISAs, and line blots purchased from EUROIMMUN Inc. (Lübeck, Germany) and evaluated by EUROIMMUN devices (Analyzer, Euroblotone, and Sprinter XL) and fluorescence microscope (IIFTs). Overall, 44 humoral AABs were measured, and seven screening tests were conducted as reported elsewhere (5). In the present study, we analyzed the results of the five most frequently detected AABs (rheumatoid factor (RF), ß2-glycoprotein (IgM), cardiolipin (IgM), cardiolipin (IgG), and anti-dsDNA) as well as all screening tests which refer to systemic rheumatic diseases (ANA nuclear, ANA cytoplasmic, ANA mitotic, ANCA, cANCA, pANCA, ENA) (see **Supplementary Table S2**). Among the excluded AABs were anti-CCP and ß2GPI IgG. Positivity of AABs was defined according to cutoffs recommended by the manufacturer for each type of blood analysis (IIFT, ELISA, or line blot) and the clinical laboratory of the University Hospital Augsburg (**Supplementary Table S2**). An exception was RF and ß2-g-glycoprotein IgM (ß2GPI). Based on the EULAR guidance (12), RF was classified as “high-positive” with values higher than three times the upper limit of normal (ULN), and ß2GPI results ≥40 U/l were defined as “moderate-positive” (13). This was carried out to increase specificity, given the high frequency of RF positivity in the sample when using the ULN value as the cut-off value. In the main analysis, the participants with (high-)positive results were compared to all other persons, called “non-positive”. In a sensitivity analysis, non-normal findings were compared to “normal” findings, where the cut-offs were lowered to 14 U/l for RF and 20 U/l for ß2GPI, and all borderline results were captured in the group “non-normal” (**Supplementary Table S2**).

The test results were not used for diagnostic purposes. Positive test results were not validated by means of other test methods.

### 2.5 Confounders

Body weight and height were measured on occasion of blood collection, and body mass index (BMI, kg/m²) was calculated. Sports activity was assessed during the baseline interview and repeated 24-h recalls (14), respectively. The concentration of plasma C-reactive protein (CRP) was analyzed in the clinical laboratory. Fatty acid analysis of RBC membranes was conducted using gas chromatography in combination with a flame ionization detector as described elsewhere (15). Fatty acids were expressed as the percentage of total fatty acid methyl esters (% FAME). Serum β-carotene concentrations (µmol/l) were estimated by high-performance liquid chromatography–ultraviolet/visible spectrophotometry (9). Eicosapentaenoic acid (EPA) and ß-carotene were shown to be inversely associated with specific IgE and/or allergic rhinitis in the BVS II study (9, 15), and thus were considered as possible confounders. The variables “smoking” (never, ex, current), “hormone replacement use” (yes/no), and a proxy variable for premenopausal vs. peri-/postmenopausal status, i.e., age ≤ 50 years and age >50 years (in women) were tested as possible confounders; as we saw no impact of all three variables on the model results, these were not included in the final models.

We did not collect information on current use of immunomodulatory drugs or corticosteroids, known autoimmune diseases, or infections in our study; thus, we are unable to consider these variables as possible confounders or for stratified analyses.

### 2.6 Statistical analysis

Characteristics of individuals with at least one positive AAB vs. non-positive persons were described as n (%) for categorical variables and as median (Q1-Q3) and arithmetic mean (standard deviation) for continuous variables. Group differences were tested with Fisher’s exact test for categorical and Mann-Whitney-U test for non-normally distributed continuous data, both within females and males.

Logistic regression models, all stratified by sex, were employed to assess the association between AAB positivity (outcome) and the two binary exposures, allergic sensitization (no/yes) and “at least one diagnosis of allergic disease” (no/yes). Only groups with acceptable numbers of persons with AAB positivity were evaluated; thus, analyses were conducted for RF, ANA nuclear, ANCA, and “at least one positive AAB test”. Also for this approach, the power was limited, and chance findings cannot be excluded.

The initial model included adjustment only for age, while the multivariable-adjusted models included BMI, sports activity (yes/no), CRP, EPA in RBC, and serum β-carotene concentration. All continuous covariables (age, BMI, CRP, EPA, β-carotene) were tested for multicollinearity with the variance inflation factor and for correlation with a correlation matrix. Statistical significance was set at p<0.05. Analyses were primarily conducted using Python with pandas (16), NumPy (17), statsmodels (18), matplotlib (19), and tableone (20) packages. In addition, R was used for validation.

## 3 Results

Characteristics of 199 female and 124 male study participants are listed in **Table 1**. On average, men were older and had a higher BMI as compared to women. In 46.2% and 37.1% of female and male study participants, respectively, at least one positive AAB test was identified. Comparing individuals (high-)positive vs. non-positive for AABs, male positives were older and showed a lower mean BMI. Allergic sensitization (specific IgE, CAP class ≥ 2) was observed in 23.8% and 29.7% of female and male participants, while 26.2% and 9.3% reported at least one diagnosis of allergic disease, respectively. Asthma, allergic rhinitis, atopic dermatitis, and food allergy were considered for the variable “at least one diagnosis of allergic disease” (**Table 1**). No distinct differences between the study sample and the full sample with blood collection existed (see **Supplementary Table S1**).

**Table 1:** Descriptive statistics of the study participants, overall and stratified by sex, and presence of at least one positive autoantibody (AAB) test in serum.

Variable	level		At least one positive AAB test			At least one positive AAB test		
			in females			in males		
			(high-) positive^#^ (1) vs. non-positive (0)			(high-)positive^#^ (1) vs. non-positive (0)		
		Overall	0	1	P-Value*	0	1	P-Value*
n		323	107 (53.8)	92 (46.2)		78 (62.9)	46 (37.1)	
Age (years)	median	45	44	44	0.927	44.5	54.5	0.08
	(Q1 - Q3)	(36.0 - 61.0)	(19.0 - 81.0)	(19.0 - 80.0)		(19.0 - 80.0)	(20.0 - 75.0)	
Sex, n (%)	w	199 (61.6)	107 (53.8)	92 (46.23)	1			
	m	124 (38.4)				78 (62.9)	46 (37.10)	1
BMI (kg/m²)	median (Q1 - Q3)	25.51 (22.8 -29.1)	24.77 (22.4 - 28.4)	25.57 (22.1 - 29.1)	0.281	26.86 (23.7 - 31.2)	25.05 (24.0 - 27.5)	0.070
Education**, n (%)	low	131 (40.6)	36 (33.6)	39 (42.4)	0.413	33 (42.3)	23 (50.0)	0.383
	middle	114 (35.3)	51 (47.7)	33 (35.9)		17 (21.8)	13 (28.3)	
	high	66 (20.4)	17 (15.9)	17 (18.5)		23 (29.5)	9 (19.6)	
	n.a.	12 (3.7)	3 (2.8)	3 (3.3)		5 (6.4)	1 (2.2)	
Sports activity, n (%)	no	178 (55.1)	60 (56.1)	54 (58.7)	0.819	38 (48.7)	26 (56.5)	0.513
	yes	145 (44.9)	47 (43.9)	38 (41.3)		40 (51.3)	20 (43.5)	
C-reactive protein (μmol/l)	median (Q1 - Q3)	1.54 (0.8 - 3.9)	1.7 (0.8 - 5.1)	1.6 (0.8 - 3.9)	0.696	1.6 (0.6 - 3.0)	1.5 (0.7 - 3.4)	0.603
RBC eicosapentaenoic acid (C20:5 n3, % FAME)	median (Q1 - Q3)	0.5 (0.9 - 1.4)	0.9 (0.6 - 1.3)	1 (0.6 - 1.5)	0.509	0.8 (0.5 - 1.5)	0.9 (0.6 - 1.3)	0.816
Serum ß-carotene (μmol/l)	median (Q1 - Q3)	1.4 (1.0 - 1.8)	1.6 (1.2 - 1.9)	1.5 (1.1 - 1.9)	0.382	1.2 (1.0 - 1.5)	1.4 (1.0 - 1.8)	0.140
Allergic sensitization (CAP class >= 2), n (%)	0	238 (73.7)	87 (81.3)	64 (69.6)	0.078	54 (69.2)	33 (71.7)	0.927
	1	85 (26.3)	20 (18.7)	28 (30.4)		24 (30.8)	13 (28.3)	
Allergic rhinitis, n (%)	0	283 (87.6)	93 (86.9)	74 (80.4)	0.295	74 (94.9)	42 (91.3)	0.467
	1	40 (12.4)	14 (13.1)	18 (19.6)		4 (5.1)	4 (8.7)	
Atopic dermatitis, n (%)	0	312 (96.6)	104 (97.2)	86 (93.5)	0.687	76 (97.4)	46 (100)	0.53
	1	11 (3.4)	3 (2.8)	6 (6.5)		2 (2.6)	0 (0)	
Food allergy, n (%)	0	305 (94.4)	100 (93.5)	82 (89.1)	0.404	77 (98.7)	46 (100)	1
	1	18 (5.6)	7 (6.5)	10 (10.9)		1 (1.3)	0 (0)	
Asthma, n (%)	0	305 (94.4)	102 (95.3)	82 (89.1)	0.113	77 (98.7)	44 (95.7)	0.555
	1	18 (5.6)	5 (4.7)	10 (10.9)		1 (1.3)	2 (4.3)	
At least one diagnosis of	0	260 (80.5)	83 (77.6)	64 (69.6)	0.257	72 (92.3)	41 (89.1)	0.535
allergic disease, n (%)	1	63 (19.5)	24 (22.4)	28 (30.4)		6 (7.7)	5 (10.9)	

*Chi²-test for categorical variables, Fisher’s exact test for categorical variables where more than 20% of cells had expected frequencies<5, t-test for continuous variables (normal distributed), Mann-Whitney-u test (MWU) for not normally distributed variables.

**education level classified into: 1, main school, elementary school, secondary school; 2, junior high school, polytechnic secondary school; 3 high school, extended secondary school.

#“high positive” refers to the classification of RF according to 3x ULN (upper limit of normal).

The number of individuals with (high-)positive test results was compared to non-positive persons, overall and stratified for the two exposure variables, allergic sensitization and ‘at least one diagnosis of allergic disease’ (**Table 2**). Among sensitized individuals, there were 12 women and 5 men who were high-positive for RF (i.e., 3xULN); other AABs with numbers sufficient for running a regression model were ANA nuclear, ANCA, and the summary estimate “at least one positive AAB test”. The same was true for the number of subjects with allergic diseases; here also, regression models for RF, ANA nuclear, ANCA, and the summary estimate were run.

**Table 2:** Frequency of allergic sensitization and at least one diagnosis of allergic disease, by systemic autoantibody (AAB) status, comparing female and male participants with (high-)positive vs. non-positive test results.

Autoantibody	Test result		Allergic sensitization				At least one diagnosis of allergic disease			
			Women		Men		Women		Men	
		Overall	no	yes	no	yes	no	yes	no	yes
		n (%)	n (%)	n (%)	n (%)	n (%)	n (%)	n (%)	n (%)	n (%)
Five most frequent AABs:										
Rheumatoid factor (RF)	non positive	266 (82.4)	128 (84.8)	36 (75.0)	70 (80.5)	32 (86.5)	129 (84.3)	35 (76.1)	94 (82.5)	8 (80.0)
	high-positive^#^	57 (17.6)	23 (15.2)	12 (25.0)	17 (19.5)	5 (13.5)	24 (15.7)	11 (23.9)	20 (17.5)	2 (20.0)
ß2-Glycoprotein IgM	non positive	316 (97.8)	146 (96.7)	48 (100)	85 (97.7)	37 (100)	149 (97.4)	45 (97.8)	112 (98.2)	10 (100)
	moderate-positive	7 (2.2)	5 (3.3)	0 (0)	2 (2.3)	0 (0)	4 (2.6)	1 (2.2)	2 (1.8)	0 (0)
Cardiolipin IgG	non positive	318 (98.5)	148 (98.0)	46 (95.8)	87 (100)	37 (100)	150 (98.0)	44 (95.7)	114 (100)	10 (100)
	positive	5 (1.5)	3 (2.0)	2 (4.2)	0 (0)	0 (0)	3 (2.0)	2 (4.3)	0 (0)	0 (0)
Cardiolipin IgM	non positive	320 (99.1)	149 (98.7)	47 (97.9)	87 (100)	37 (100)	151 (98.7)	45 (97.8)	114 (100)	10 (100)
	positive	3 (0.9)	2 (1.3)	1 (2.1)	0 (0)	0 (0)	2 (1.3)	1 (2.2)	0 (0)	0 (0)
Anti-dsDNA	non positive	319 (98.8)	149 (98.7)	48 (100)	85 (97.7)	37 (100)	151 (98.7)	46 (100)	112 (98.2)	10 (100)
	positive	4 (1.2)	2 (1.3)	0 (0)	2 (2.3)	0 (0)	2 (1.3)	0 (0)	2 (1.8)	0 (0)
AAB screening tests:										
ANA nuclear	non positive	284 (87.9)	134 (88.7)	37 (77.1)	78 (89.7)	35 (94.6)	132 (86.3)	39 (84.8)	104 (91.2)	9 (90.0)
	positive	39 (12.1)	17 (11.3)	11 (22.9)	9 (10.3)	2 (5.4)	21 (13.7)	7 (15.2)	10 (8.8)	1 (10)
ANA cytoplasmic	non positive	313 (96.9)	146 (96.7)	45 (93.8)	85 (97.7)	37 (100)	147 (96.1)	44 (95.7)	112 (98.2)	10 (100)
	positive	10 (3.1)	5 (3.3)	3 (6.3)	2 (2.3)	0 (0)	6 (3.9)	2 (4.3)	2 (1.8)	0 (0)
ANA mitotic	non positive	318 (98.5)	149 (98.7)	46 (95.8)	86 (98.9)	37 (100)	151 (98.7)	44 (95.7)	113 (99.1)	10 (100)
	positive	5 (1.5)	2 (1.3)	2 (4.2)	1 (1.1)	0 (0)	2 (1.3)	2 (4.3)	1 (0.9)	0 (0)
ANCA	non positive	273 (84.5)	126 (83.4)	39 (81.3)	78 (89.7)	30 (81.1)	125 (81.7)	40 (87.0)	99 (86.8)	9 (90.0)
	positive	50 (15.5)	25 (16.6)	9 (18.8)	9 (10.3)	7 (18.9)	28 (18.3)	6 (13.0)	15 (13.2)	1 (10)
cANCA	non positive	322 (99.7)	150 (99.3)	48 (100)	87 (100)	37 (100)	152 (99.3)	46 (100)	114 (100)	10 (100)
	positive	1 (0.3)	1 (0.7)	0 (0)	0 (0)	0 (0)	1 (0.7)	0 (0)	0 (0)	0 (0)
pANCA	non positive	306 (94.7)	143 (94.7)	43 (89.6)	84 (96.6)	36 (97.3)	141 (92.2)	45 (97.8)	110 (96.5)	10 (100)
	positive	17 (5.3)	8 (5.3)	5 (10.4)	3 (3.4)	1 (2.7)	12 (7.8)	1 (2.2)	4 (3.5)	0 (0)
ENA-Screening	non positive	318 (98.5)	149 (98.7)	46 (95.8)	87 (100)	36 (97.3)	152 (99.3)	43 (93.5)	113 (99.1)	10 (100)
	positive	5 (1.5)	2 (1.3)	2 (4.2)	0 (0)	1 (2.7)	1 (0.7)	3 (6.5)	1 (0.9)	0 (0)
At least one (high-) positive AAB test	non positive	185 (57.3)	87 (57.6)	20 (41.7)	54 (62.1)	24 (64.9)	86 (56.2)	21 (45.7)	72 (63.2)	6 (60.0)
	positive	138 (42.7)	64 (42.4)	28 (58.3)	33 (37.9)	13 (35.1)	67 (43.8)	25 (54.3)	42 (36.8)	4 (40.0)

#“high-positive” refers to the classification of RF according to 3x ULN (upper limit of normal).

Positive associations with odds ratios (ORs) between 2.0 and 2.8 between allergic sensitization and serum AABs were identified in women for RF, nuclear ANA, and at least one positive AAB test (**Table 3**). In women, allergic sensitization increased the chance for the presence of RF (OR 2.19; 95% CI 0.94-5.11; p=0.069). With an OR (95% CI) of 2.85 (1.15-7.05) (p=0.023), the association was statistically significant for nuclear ANA and close to significance for “at least one positive AAB test” (1.99 (0.996-3.98), p=0.051). In men, for the association of ANCA and allergic sensitization, an elevated OR of 2.88 (0.84-9.89) was observed but did not reach statistical significance (p=0.092).

**Table 3:** Odds ratio (OR) and 95% confidence interval (95% CI) for the association between allergic sensitization (yes/no; exposure) and serum autoantibodies ((high-)positive^#^ vs. non-positive; outcomes), stratified by sex.

AABs	Sex	Raw model*				Extended model**			
		OR	95% CI		P-value	OR	95% CI		P-value
Rheumatoid factor (RF)	f	1.963	0.871	4.422	0.104	2.192	0.941	5.107	0.069
	m	0.708	0.218	2.146	0.541	0.603	0.190	1.921	0.393
ANA nuclear	f	2.343	1.010	5.435	0.047	2.851	1.153	7.051	0.023
	m	0.495	0.102	2.412	0.384	0.388	0.072	2.075	0.268
ANCA	f	1.163	0.501	2.700	0.725	0.932	0.375	2.318	0.880
	m	2.022	0.691	5.918	0.199	2.879	0.841	9.855	0.092
At least one (high-)positive AAB test	f	1.953	0.996	3.830	0.051	1.991	0.996	3.978	0.051
	m	1.061	0.459	2.339	0.890	0.903	0.361	2.263	0.828

*adjusted for age.

**adjusted for age, BMI, sport (yes/no), CRP, EPA, ß-carotene.

#“high-positive” refers to the classification of RF according to 3x ULN (upper limit of normal).

In the sensitivity analysis, study participants with normal AAB test values were compared to persons with non-normal results, including all test results between the classifications ‘normal’ and ‘positive’. The descriptive data are given in the **Supplementary Tables S3** and **S4**. The results of the logistic regression analysis for the association of allergic sensitization with RF became statistically significant with an OR = 2.16 (1.08-4.33, p=0.030), and the association with “at least one non-normal AAB test” became stronger (3.17 (1.37-7.34), p=0.007) than observed in the main analysis (**Table 4**). Comparing different definitions of allergic sensitization (CAP ≥ 0.70 or CAP ≥ 0.35) showed similar results and thus allowed the same conclusions (**Supplementary Table S6**).

**Table 4:** Sensitivity analysis: Odds ratio (OR) and 95% confidence interval (95% CI) for the association between allergic sensitization (yes/no; exposure) and serum autoantibodies (non-normal vs. normal; outcomes), stratified by sex.

AABs	Sex	Raw model*				Extended model**			
		OR	95% CI		P-value	OR	95% CI		P-value
RF	f	2.020	1.045	3.906	**0.037**	2.157	1.076	4.325	**0.030**
	m	1.283	0.575	2.861	0.543	1.190	0.489	2.896	0.702
ANA nuclear	f	1.247	0.639	2.435	0.517	1.262	0.625	2.548	0.517
	m	0.754	0.313	1.818	0.530	0.724	0.281	1.868	0.504
ANCA	f	1.163	0.501	2.700	0.725	0.932	0.375	2.318	0.880
	m	2.022	0.691	5.918	0.199	2.879	0.841	9.855	0.092
At least one non-normal AAB test	f	2.857	1.291	6.325	**0.010**	3.168	1.367	7.342	**0.007**
	m	0.987	0.451	2.162	0.974	1.012	0.427	2.401	0.978

*adjusted for age.

**adjusted for age, BMI, sport (yes/no), CRP, EPA, ß-carotene.

bold letters show significant values where p <= 0.5

Regarding the diagnosis of allergic diseases, the results of the multivariable-adjusted regression models were not statistically significant with both definitions, positive vs. non-positive (**Table 5**), and non-normal vs normal (**Supplementary Table S5**).

**Table 5:** Odds ratio (OR) and 95% confidence interval (95% CI) for the association between at least one diagnosis of allergic disease (yes/no; exposure) and serum autoantibodies ((high-)positive^#^ vs. non-positive; outcome), stratified by sex.

AABs	Sex	Raw model*				Extended model**			
		OR	95% CI		P-value	OR	95% CI		P-value
Rheumatoid factor (RF)	f	1.635	0.745	3.589	0.220	1.954	0.859	4.447	0.110
	m	1.054	0.210	5.293	0.949	0.879	0.115	4.994	0.884
ANA nuclear	f	1.154	0.474	2.813	0.752	1.221	0.483	3.085	0.674
	m	2.662	0.490	14.463	0.257	1.942	0.284	13.258	0.498
ANCA	f	0.673	0.273	1.660	0.390	0.704	0.275	1.803	0.465
	m	0.665	0.079	5.622	0.708	0.385	0.036	4.068	0.427
At least one (high-)positive AAB test	f	1.515	0.801	2.864	0.201	1.702	0.873	3.319	0.118
	m	1.532	0.430	5.458	0.510	1.105	0.271	4.508	0.890

*adjusted for age.

**adjusted for age, BMI, sport (yes/no), CRP, EPA, ß-carotene.

#“high-positive” refers to the classification of RF according to 3x ULN (upper limit of normal).

## 4 Discussion

In this population-based study, we observed associations between allergic sensitization and positivity or non-normality for serum AAB or screening tests in women. Significantly increased odds ratios for RF, nuclear ANA, and at least one positive or non-normal AAB test were obtained. Regarding allergic diseases, no association reached statistical significance.

The prevalence of (at least one) positive AAB test in our study population is high compared to the results of the still rare population-based studies. After analyzing 5 circulating AABs and 7 screening tests in the present study, the overall prevalence was 46.3% and 37.1% in women and men, respectively. In the NHANES study, eight individual AABs were analyzed in a representative sample of the U.S. population, and a prevalence of 39% in women and 22% in men was reported (1). The overlap in both studies was limited, as only RF, nuclear ANA, and ENA were analyzed in both studies. The most frequently found AABs in the NHANES study were anti-thyroglobulin, anti-thyroperoxidase, anti-tissue transglutaminase, and ANA. In the present study, the five most frequent AABs and the seven screening tests are listed in **Table 2**. Besides RF, the more frequently found AABs comprise ß2-glycoprotein (IgM), cardiolipin (IgM), cardiolipin (IgG), and anti-dsDNA. All screening tests refer to systemic rheumatic diseases (ANA nuclear, ANA cytoplasmic, ANA mitotic, ANCA, cANCA, pANCA, ENA). The comparison with the NHANES AAB panel demonstrates that there are many more AABs than considered in the present study. In the following, the focus is on those AAB tests that were most frequently determined and thus could be examined for an association with allergic sensitization of allergies, i.e., RF, ANA, ANCA, and the summary variable “at least one positive AAB test”.

The prevalence of allergic rhinitis and allergic sensitization in our study is in good agreement with published data at a comparable calendar time (21, 22). A prevalence of 33.6% for the sensitization to common aeroallergens was reported for a random sample of German adults in the DEGS1 study conducted between 2008 and 2011 (23), which is higher than the here observed prevalence. However, men were more frequently sensitized to at least one allergen than women. In our study, distinctly more women than men participated in the full study and the same is true for the present study sample (see also **Supplementary Table S1** for comparison).

The prevalence of allergic diseases in German adults was reported in the same study with lifetime prevalences of 8.6% for asthma (5.6% in the present study), 14.8% for allergic rhino-conjunctivitis (12.4% in the present study), 3.5% for atopic dermatitis (3.4% in the present study), and 4.7% for food allergies (5.6% in the present study) (24). In men, figures were distinctly lower for asthma and allergic rhinitis than reported in DEGS1.

Allergic hypersensitivity is initiated by immunological mechanisms dependent on antibodies that belong to the IgE class of immunoglobulins. IgE-dependent mechanisms were observed in asthma, allergic rhinitis, atopic dermatitis, and some forms of urticaria. Environmental factors associated with the presence of allergens that cause immune hypersensitivity may also affect and condition the development of AIDs (3).

### 4.1 RF

Systemic connective tissue diseases are a classic example of AIDs that develop as a result of immune tolerance disorders. The presence of AABs in blood and joint fluid is a characteristic feature of rheumatoid arthritis (RA) that distinguishes this disease from other inflammatory joint disorders. The hallmark antibodies of RA are RF and anticitrullinated protein antibodies (ACPA), which are detectable in 60–70% of RA patients already in the earliest stages of the disease (25). The RF is a representative AAB against the crystallizable fragment (Fc) of denatured IgG that is primarily detected in patients with RA (26). RF is not limited to RA but is also found in patients with other AIDs like Sjögren’s syndrome, or infectious diseases like hepatitis and tuberculosis, as well as in non-symptomatic healthy individuals. In 2010, the American College of Rheumatology/European League Against Rheumatism developed new classification criteria for RA, including serologic markers. They suggested defining high-positive RF values, i.e., values exceeding ULN by more than three times (12). We followed this suggestion and ended with 17.6% of our population classified as high-positive. However, when applying the definition of values above normal (RF≥14 U/l), 35.9% of our population had non-normal RF values, meaning that 1/3 of the population has detectable RF. In our study, RF positivity in women was associated with allergic sensitization and reached statistical significance when using the non-normality definition (OR = 2.16, p<0.030). However, a relationship with allergic diseases was only observed in women when using the classification of high-positive values (versus other values) with an OR of 2.50 (p<0.026).

This finding is supported by several systematic reviews and meta-analyses (SR&MA) that examined the association between allergic diseases and RA. One SR&MA showed a significantly higher risk of incident RA in atopic dermatitis patients (27). Subgroup analysis also revealed a significantly higher risk of RA in cohort studies (pooled OR, 1.37; 95% CI, 1.25–1.50). In another SR&MA, a significantly higher risk of RA among patients with allergic rhinitis than individuals without allergic rhinitis was observed when only studies with acceptable quality were included (28). A third SR&MA described a significant association between asthma and a higher risk of incident RA but found evidence of publication bias (29). Patients with asthma were also at higher risk of systemic lupus erythematosus (30). A large population-based study in the U.S. (NHANES) with 20,050 participants found that physician diagnosis of allergic disorders was associated with an increased risk of physician-diagnosed AIDs (adjusted odds ratio, 1.67; 95% CI, 1.35-2.07; p<0.001) (31). However, studies investigating direct associations between RF and allergic diseases or RF and allergic sensitization could not be identified (32).

### 4.2 ANA

The confirmation of ANA positivity in a healthy individual is usually of unknown significance and, in most cases, benign. From a clinical point of view, the classic screening test for SLE is the presence in serum of ANAs, and ANA positivity is required to make a diagnosis of lupus since more than 99% of patients with SLE have significant levels of this ABB detected at some time during the course of the disease. However, since the prevalence of SLE is low, most individuals presenting to a physician with ANA positivity do not have SLE and are not at high risk for developing this disease (2, 3).

The percentage of the population with ANAs was reported to be approximately 25% by using the standard analysis methods (indirect immunofluorescence assay performed on HEp-2 cells (IIFA on HEp-2 or HEp-2000)), and 2.5% have distinctly elevated ANA levels (2, 33). According to other reports using IIFA, ANA in low counts may appear in up to 40% of healthy people (3, 34), which is in good agreement with the here observed ANA prevalence of 42% with non-normal values (**Supplementary Table S4**); also, the prevalence of 17% of individuals with positive results (**Table 2**) in our study fits well with the prevalence of 14% measured in the U.S. population (1, 35) or 15.8% obtained in a Swedish population (36). Among the NHANES participants who had ANA, nuclear staining was seen in 85%. ANA positivity is more frequently detected in women, and thus female gender is a risk factor for ANA positivity (3, 35). Both the high percentage of nuclear ANA and the female dominance were confirmed in our study (**Table 2**).

The relationship between ANA occurrence and allergic diseases is poorly documented. However, the mechanism of allergic sensitization and AIDs has a common thread. An increased production of IgE antibodies and the presence of ANA in selected disease entities were observed. Both autoimmune and allergic diseases seem to show overlapping pathogenic processes and often occur in genetically predisposed individuals (3). The activation of basophils secreting proinflammatory factors and affecting the differentiation of TH17 lymphocytes occurs in both conditions. The presence of ANAs was confirmed in many systemic connective tissue diseases and some allergic diseases, such as atopic dermatitis, non-allergic asthma, and pollen allergy (3). In the present study, we found a strong positive association between nuclear ANA positivity and allergic sensitization (OR = 2.85, p<0.023) only in women, but no association with allergic diseases, arguing for a relationship between joint biologic processes rather than between diseases.

### 4.3 ANCA

ANCA-associated vasculitis (AAV) is a disease entity characterized by systemic vasculitis positive for ANCA; it often leads to severe organ damage, such as diffuse bronchoalveolar hemorrhage and rapidly progressive glomerulonephritis (37). ANCA targets lysosomal proteins, such as proteinase 3, myeloperoxidase, and lactoferrin. The presence of ANCA was also reported in other diseases such as inflammatory bowel disease (38) and SLE (39). It is more frequently found in males than females (39), which was confirmed in the present study. The literature describes several situations and diseases related to ANCA positivity (AAV) where an association with allergies has been identified. For example, in eosinophilic granulomatosis with polyangiitis (EGPA), nearly all patients have an allergic background (asthma and/or allergic sinusitis) (40). However, we could not identify a population-based study that explored the relationship between ANCA positivity (without clinical diagnoses) and allergic sensitization or allergic disease. The increased OR observed for the association between ANCA positivity and allergic sensitization in men did not reach statistical significance, and no association was seen with allergic diseases.

### 4.4 Summary estimate ‘at least one positive AAB test’

Due to their presence in the general population as well as in multiple AIDs, the presence of an AAB alone does not make a diagnosis; the result has to be interpreted along with clinical findings. Similarly, the absence of AABs does not exclude a disease (41). The common AABs used in clinical practice include RF, anti-CCP antibodies, ANAs, ANCA, and antiphospholipid antibodies. These and other AABs were included in the variable “at least one positive AAB test”. Thus, this variable reflects the presence of biologic autoimmunity in individuals. Testing its association with allergic sensitization means searching for an association with biologic allergenicity. Overall, we obtained evidence for an association between these two biological phenotypes. Accordingly, this association was strongest (OR = 3.17) and statistically significant (p<0.007) after including all non-normal findings (**Table 4**), and no association with allergic disease was observed.

Both allergic and autoimmune diseases are marked by a dysregulation of the immune system, reacting to otherwise harmless environmental substances or autoantigens, which leads to inflammation and tissue damage (42). Allergies are primarily driven by type 2 inflammation, which can intensify autoimmune responses in susceptible individuals by promoting tissue damage or autoantibody production. The type 2 immune response, mainly triggered by the activation of type 2 helper T cells (Th2 cells), plays a central role in the overlap between allergies and certain autoimmune conditions due to shared immune pathways, the release of specific cytokines (signaling molecules), and the activation of immune cells that drive processes like eosinophil activation, antibody production, and tissue repair (43). However, other factors, including genetic predisposition, environmental triggers, and additional immune mechanisms (e.g., Th1/Th17 responses or autoantibodies), also play significant roles (42). The interplay is intricate and varies depending on the specific combination of diseases.

The association between allergies and autoimmune diseases, observed to be stronger in women in this study, likely arises from a combination of hormonal, genetic, and environmental factors that differentially regulate immune responses by sex (44). In women, estrogen amplifies Th2 (allergy-related) and Th17 (autoimmunity-related) signaling pathways (45, 46). Conversely, in men, testosterone suppresses Th2 and Th17 responses, promotes regulatory T cells (Tregs), reducing inflammation and the overlap between these conditions (47).

The odds ratio for ANA nuclear and allergic sensitization was statistically significant when comparing high-positive vs. non-positive, but no association existed when comparing non-normal vs. normal. The opposite was observed with RF. As this resulted only from the change in classification, these results must be interpreted as its direct consequence. When including non-normal values in the evaluation, the associations became slightly stronger for RF and for the summary variable “at least one non-normal AAB test”. The results of the summary variable seem to be driven by RF, leading to the conclusion that the results of this study support the idea of an association between AAB prevalence and allergic sensitization. However, ANA nuclear behaved differently. For RF, continuous values were measured, while for ANA nuclear different titers were used. Including the titer =1:100 as non-normal titers for ANA nuclear may have added more “noise” than clear information and thus including this titer may have not been useful as discussed by others (48).

### 4.4 Strengths and limitations

The present study investigated a comprehensive panel of systemic AABs and screening tests in the general population for their association with allergic sensitization and physician-diagnosed allergic diseases. However, many more known systemic AABs could not be included in our study. With the current sample size, only the most frequent AABs and a summary variable (at least one positive AAB test) could be evaluated in multivariable adjusted regression models. Only a very limited number of individuals were positive for more than two AAB, thus precluding the use of a continuous variable “number of AAB”. There is an increased risk of chance findings due to the limited number of positive subjects for some individual AABs, and thus underpowered analyses. Due to the requirement to use unthawed samples for AAB analysis, we could include only 331 subjects (out of a study with 568 subjects).

Also, the exposure variables (specific IgE, diagnosis of allergy) are either not comprehensive or low in numbers. The Phadiatop test (CAPSX1) analyzed antibodies (specific IgE) against the most common antigens, while many more antigens are known. Among allergic diseases, we could not assess all atopic diseases or all type-1 allergies.

Due to the cross-sectional design of our study, our findings cannot be used to infer causality. In addition, the specific features of the Bavarian population sample preclude a direct transfer of the findings to other ethnicities and age groups. We did not collect information on current use of immunomodulatory drugs or corticosteroids, known autoimmune diseases, or infections in our study; thus, unmeasured confounding cannot be excluded. In addition, analytic results may vary depending on the manufacturer’s test design and test performance, which could explain some differences in frequencies observed for certain AABs in different studies. Notably, all test kits used in our analyses were purchased from the same provider and analyzed in one laboratory.

## 5 Conclusion

Our study is one of the first population-based studies exploring a link between a large panel of systemic AABs and allergic sensitization or allergic diseases. The findings support the idea that there is a link between biological autoimmunity and allergic sensitization, as associations with allergic diseases were not identified. The observed link between the two biological phenotypes seems to be limited to female participants. Further studies should investigate larger population samples with sufficiently high numbers of AAB-positive individuals and use a prospective study design.

## Data Availability

The raw data supporting the conclusions of this article will be made available by the authors, without undue reservation.
