# Supplementary material for: Sex-specific associations between systemic autoantibodies and allergic sensitization or allergic disease – results from a population-based study
Source: Front Immunol. 2026 Jan 16;16:1740193. doi: 10.3389/fimmu.2025.1740193 (PMC12855144; doi:10.3389/fimmu.2025.1740193)
Supplement: Supplementary file 1 [file DataSheet1.docx]

**Supplementary Tables**

**Table S1: Comparison of characteristics of the current study population with all study participants with blood samples available**

|  |  | **Participants with AAB-screening  (n=331)** | | | **Participants of blood collection (n=568)** | | |
| --- | --- | --- | --- | --- | --- | --- | --- |
| **Variable** |  | **Total** | **Female** | **Male** | **Total** | **Female** | **Male** |
|  |  |  | **N (%)** |  |  | **N (%)** |  |
| Level of education, n(%) | low | 133 (40.2%) | 75 (37.1%) | 58 (45.0%) | 253 (45.1%) | 134 (41.4%) | 119 (50.2%) |
|  | mid | 111 (33.5%) | 84 (41.6%) | 27 (20.9%) | 170 (30.3%) | 121 (37.3%) | 49 (20.7%) |
|  | high | 71 (21.5%) | 36 (17.8%) | 35 (27.1%) | 118 (21.0%) | 58 (17.9%) | 60 (25.3%) |
|  | unknown | 16 (4.8%) | 7 (3.5%) | 9 (7.9%) | 20 (3.6%) | 11 (3.4%) | 9 (3.8%) |
|  |  |  |  |  |  |  |  |
| Smoking status, n(%) | never | 182 (55.0%) | 126 (62.4%) | 56 (43.4%) | 292 (51.5%) | 195 (60.2%) | 97 (39.9%) |
|  | former | 66 (19.9%) | 35 (17.3%) | 31 (24.0%) | 135 (23.8%) | 61 (18.8%) | 74 (30.5%) |
|  | current | 83 (25.1%) | 41 (20.3%) | 42 (32.6%) | 140 (24.7%) | 68 (21.0%) | 72 (29.6%) |
|  |  |  |  |  |  |  |  |
| Sports activity, n(%) | no | 182 (55.0%) | 116 (57.4%) | 66 (51.2%) | 290 (51.1%) | 169 (52.0%) | 121 (49.8%) |
|  | yes | 149 (45.0%) | 86 (42.6%) | 63 (48.8%) | 278 (48.9%) | 156 (48.0%) | 122 (50.2%) |
|  |  |  |  |  |  |  |  |
| Asthma, n(%) | no | 312 (94.3%) | 187 (92.6%) | 125 (96.9%) | 534 (94.0%) | 300 (92.3%) | 234 (96.3%) |
|  | yes | 18 (5.4%) | 15 (7.4%) | 3 (2.3%) | 33 (5.8%) | 25 (7.7%) | 8 (3.3%) |
|  | unknown | 1 (0.3%) | 0 (0.0%) | 1 (0.8%) | 1 (0.2%) | 0 (0.0%) | 1 (0.4%) |
|  |  |  |  |  |  |  |  |
| Allergic rhinitis, n(%) | no | 290 (87.6%) | 169 (83.7%) | 121 (93.8%) | 491 (86.4%) | 268 (82.5%) | 223 (91.8%) |
|  | yes | 41 (12.4%) | 33 (16.3%) | 8 (6.2%) | 77 (13.6%) | 57 (17.5%) | 20 (8.2%) |
|  |  |  |  |  |  |  |  |
| Atopic dermatitis, n(%) | no | 318 (96.1%) | 192 (95.0%) | 126 (97.7%) | 545 (96.0%) | 307 (94.5%) | 238 (97.9%) |
|  | yes | 12 (3.6%) | 9 (4.5%) | 3 (2.3%) | 22 (3.9%) | 17 (5.2%) | 5 (2.1%) |
|  | unknown | 1 (0.3%) | 1 (0.5%) | 0 (0.0%) | 1 (0.2%) | 1 (0.3%) | 0 (0.0%) |
|  |  |  |  |  |  |  |  |
| Food allergy, n(%) | no | 313 (94.6%) | 185 (91.6%) | 128 (99.2%) | 536 (94.4%) | 297 (91.4%) | 239 (98.4%) |
|  | yes | 18 (5.4%) | 17 (8.4%) | 1 (0.8%) | 32 (5.6%) | 28 (8.6%) | 4 (1.6%) |
|  |  |  |  |  |  |  |  |
| Allergic sensitization (CAP class >= 2), n(%) | no | 244 (73.9%) | 154 (76.2%) | 90 (70.3%) | 416 (73.5%) | 245 (75.6%) | 171 (70.7%) |
|  | yes | 86 (26.1%) | 48 (23.8%) | 38 (29.7%) | 150 (26.5%) | 79 (24.4%) | 71 (29.3%) |
|  |  |  |  |  |  |  |  |
| At least one diagnosis of an allergic | no | 266 (80.4%) | 149 (73.8%) | 118 (90.7%) | 453 (79.8%) | 238 (73.2%) | 215 (88.5%) |
| Disease, n(%) | yes | 65 (19.6%) | 53 (26.2%) | 12 (9.3%) | 115 (20.2%) | 87 (26.8%) | 28 (11.5%) |
|  |  |  |  |  |  |  |  |
| Age (years) | median  (Q1–Q3) | 46.0  (36.0–61.0) | 44.0  (36.0–57.8) | 52.0  (37.0–63.0) | 46.0  (37.0–62.0) | 44.0  (36.0–57.0) | 53.0  (39.0–64.0) |
|  |  |  |  |  |  |  |  |
| BMI (kg/m²) | median  (Q1–Q3) | 25.6  (22.8–29.0) | 25.1  (22.4–28.8) | 26.2  (24.0–29.7) | 26.1  (23.1–29.3) | 25.3  (22.5–28.9) | 26.9  (24.3–29.7) |
|  |  |  |  |  |  |  |  |
| Hs-CRP (µmol/l) | median  (Q1–Q3) | 1.6  (0.8–3.9) | 1.7  (0.8–4.1) | 1.5  (0.7–3.3) | 1.7  (0.8–4.0) | 1.7  (0.8–4.0) | 1.7  (0.9–4.0) |
|  |  |  |  |  |  |  |  |
| RBC eicosapentaenoic acid  (C20:5 w3; % FAME) | median  (Q1–Q3) | 0.9  (0.6–1.4) | 1.0  (0.6–1.4) | 0.9  (0.5–1.4) | 0.8  (0.4–1.3) | 0.8  (0.4–1.3) | 0.7  (0.4–1.3) |
|  |  |  |  |  |  |  |  |
| Serum  ß-carotene (µmol/l) | median  (Q1–Q3) | 1.4  (1.0–1.8) | 1.5  (1.1–1.9) | 1.3  (1.0–1.7) | 1.4  (1.0–1.8) | 1.5  (1.1–2.0) | 1.2  (0.9–1.6) |

*education level classified: 1, main school, elementary school, secondary school; 2, junior high school, polytechnic secondary school; 3, high school, extended secondary school

**Table S2:** **Cut-offs for classification of autoantibody (AAB)test results as (high-)positive or non-normal**

| AAB | Lab test category | KIT name | Order number | Conjugate | Cut-off for test result | |
| --- | --- | --- | --- | --- | --- | --- |
|  |  |  |  |  | (high-)positive | non-normal |
| Rheumatoid factor (RF) | ELISA quantitativ | Rheumafaktor-ELISA (IgM) | EA 1814-9601 M | anti-Human-IgM (goat) | ≥42U/ml (3x14U/ml) | ≥14U/ml |
| ß2 Glycoprotein IgM | ELISA quantitativ | Anti-b2-Glykoprotein-ELISA (IgM) | EA 1632-9601 M | anti-Human-IgM (goat) | ≥40 U/ml | ≥20 U/ml |
| Cardiolipin IgG | ELISA quantitativ | Anti-Cardiolipin-ELISA (IgG) | EA 1621-9601 M | anti-Human-IgG (rabbit) | >12 PL-IgM-E/ml | ≥12 PL-IgM-E/ml |
| Cardiolipin IgM | ELISA quantitativ | Anti-Cardiolipin-ELISA (IgG) | EA 1621-9601G | anti-Human-IgM (goat) | >12 PL-IgM-E/ml | ≥12 PL-IgM-E/ml |
| Anti-dsDNS | ELISA quantitativ | Anti-dsDNS-ELISA (IgG) | EA 1572-901G | anti-Human-IgG (rabbit) | >100 IU/ml | ≥100 IU/ml |
| ENA-Screening | ELISA quantitativ | Anti-ENA-PoolPlus ELISA (IgG) | EA-1590-9601-7G | anti-Human-IgG (rabbit) | "positive", +++ | "positive", +++, ++ |
| ANA nuclear | IFA | HEp2-cells / liver | FA 1512-2010 | anti-Human-IgG (goat) | titer >1:100 | ≥1:100 |
| ANA cytoplasmic | IFA | HEp2-cells / liver | FA 1512-2010 | anti-Human-IgG (goat) | titer >1:100 | ≥1:100 |
| ANA mitotic | IFA | HEp2-cells / liver | FA 1512-2010 | anti-Human-IgG (goat) | titer >1:100 | ≥1:100 |
| ANCA | IFA | Granulozcyte-Mosaik | FA 1200-1010 | anti-Human-IgG (goat) | "positive" | "positive" |
| cANCA | IFA | Granulozcyte-Mosaik | FA 1200-1010 | anti-Human-IgG (goat) | titer >1:10 | ≥ 1:10 |
| pANCA | IFA | Granulozcyte-Mosaik | FA 1200-1010 | anti-Human-IgG (goat) | titer >1:10 | ≥ 1:10 |

**Table S3:** **Descriptive statistics of the study participants, overall and stratified by sex, and presence of at least one non-normal autoantibody (AAB) test in serum.**

|  |  |  | **At least one AAB test** | | | **At least one AAB test** | | |
| --- | --- | --- | --- | --- | --- | --- | --- | --- |
|  |  |  | **females** | | | **males** | | |
|  |  |  | **non-normal (1) vs. normal (0)** | | | **non-normal (1) vs. normal (0)** | | |
|  |  | **Overall** | **0** | **1** | **P-Value*** | **0** | **1** | **P-Value*** |
| **n** |  | 323 | 69 | 130 |  | 50 | 74 |  |
| **Age (years)** | **median** | 45 | 45 | 44 | 0.645 | 51 | 51 | 0.838 |
|  | **(Q1 - Q3)** | (36.0 - 61.0) | (37.0 - 59.0) | (35.2 - 57.0) |  | (35.2 - 62.0) | (37.2 - 62.8) |  |
| **Sex, n (%)** | **w** | 199 (61.6) | 69 (100.0) | 130 (100.0) | 1 |  |  |  |
|  | **m** | 124 (38.4) |  |  |  | 50 (100.0) | 74 (100.0) | 1 |
| **BMI (kg/m²)** | **median**  **(Q1 - Q3)** | 25.51  (22.8 -29.1) | 24.5  (22.4 - 28.7) | 25.3  (22.3 - 28.9) | 0.364 | 27.5  (24.6 - 31.0) | 25.4  (23.5 - 28.7) | 0.05 |
| **Education**, n (%)** | **low** | 131 (40.6) | 23 (33.3) | 52 (40.0) | 0.501 | 20 (40.0) | 36 (48.6) | 0.397 |
|  | **middle** | 114 (35.3) | 33 (47.8) | 51 (39.2) |  | 14 (28.0) | 16 (21.6) |  |
|  | **high** | 66 (20.4) | 10 (14.5) | 24 (18.5) |  | 15 (30.0) | 17 (23.0) |  |
|  | **n.a** | 12 (3.7) | 3 (4.3) | 3 (2.3) |  | 1 (2.0) | 5 (6.8) |  |
| **Sports activity, n (%)** | **no** | 178 (55.1) | 39 (56.5) | 75 (57.7) | 0.993 | 29 (58.0) | 35 (47.3) | 0.324 |
|  | **yes** | 145 (44.9) | 30 (43.5) | 55 (42.3) |  | 21 (42.0) | 39 (52.7) |  |
| **C-reactive protein**  **(μmol/l)** | **median**  **(Q1 - Q3)** | 1.54  (0.8 - 3.9) | 1.7  (0.8 - 7.0) | 1.6  (0.8 - 3.8) | 0.362 | 1.6  (0.6 - 3.9) | 1.3  (0.8 - 3.2) | 0.825 |
| **Serum eicosapentaenoic acid**  **(C20:5 n3, % FAME)** | **median**  **(Q1 - Q3)** | 0.5  (0.9 - 1.4) | 1.1  (0.6 - 1.4) | 0.9  (0.5 - 1.4) | 0.277 | 0.9 | 0.9 | 0.953 |
|  |  |  |  |  |  | (0.5 - 1.5) | (0.5 - 1.3) |  |
| **Serum ß-carotene (μmol/l)** | **median**  **(Q1 - Q3)** | 1.4  (1.0 - 1.8) | 1.6  (1.2 - 1.9) | 1.6  (1.1 - 2.0) | 0.873 | 1.2 | 1.3 | 0.07 |
|  |  |  |  |  |  | (0.9 - 1.5) | (1.0 - 1.8) |  |
| **Allergic sensitization (CAP class >= 2),** | **0** | 238 (73.7) | 60 (87.0) | 91 (70.0) | **0.013** | 35 (70.0) | 52 (70.3) | 1 |
| **n (%)** | **1** | 85 (26.3) | 9 (13.0) | 39 (30.0) |  | 15 (30.0) | 22 (29.7) |  |
| **Allergic rhinitis** | **0** | 283 (87.6) | 58 (84.1) | 109 (83.8) | 1 | 47 (94.0) | 69 (93.2) | 1 |
|  | **1** | 40 (12.4) | 11 (15.9) | 21 (16.2) |  | 3 (6.0) | 5 (6.8) |  |
| **Atopic dermatitis** | **0** | 312 (96.6) | 68 (98.6) | 122 (93.8) | 0.167 | 48 (96.0) | 74 (100) | 0.161 |
|  | **1** | 11 (3.4) | 1 (1.4) | 8 (6.2) |  | 2 (4.0) | 0 (0) |  |
| **Food allergy** | **0** | 305 (94.4) | 65 (94.2) | 117 (90.0) | 0.427 | 49 (98.0) | 74 (100) | 0.403 |
|  | **1** | 18 (5.6) | 4 (5.8) | 13 (10.0) |  | 1 (2.0) | 0 (0) |  |
| **Asthma** | **0** | 305 (94.4) | 66 (95.7) | 118 (90.8) | 0.269 | 49 (98.0) | 72 (97.3) | 0.735 |
|  | **1** | 18 (5.6) | 3 (4.3) | 12 (9.2) |  | 1 (2.0) | 2 (2.7) |  |
| **At least one diagnosis of** | **0** | 260 (80.5) | 54 (78.3) | 93 (71.5) | 0.397 | 45 (90.0) | 68 (91.9) | 0.755 |
| **an allergic disease** | **1** | 63 (19.5) | 15 (21.7) | 37 (28.5) |  | 5 (10.0) | 6 (8.1) |  |

| *Chi²-test for categorical variables, Fisher’s exact test for categorical variables where more than 20% of cells had expected frequencies <5, t-test for continuous variables (normally distributed), Mann-Whitney-u test (MWU) for not normally distributed variables |  |  |  |  |  |  |  |  |  |
| --- | --- | --- | --- | --- | --- | --- | --- | --- | --- |
| **education level classified: 1, main school, elementary school, secondary school; 2, junior high school, polytechnic secondary school; 3, high school, extended secondary school | |  |  |  |  |  |  |  |  |

**Table S4: Frequency of allergic sensitization and at least one diagnosis of allergic disease, by systemic autoantibody (AAB) status, comparing female and male participants with non-normal vs. normal test results**

|  |  |  | **Allergic sensitization** | | | | **At least one diagnosis of allergic disease** | | | |
| --- | --- | --- | --- | --- | --- | --- | --- | --- | --- | --- |
|  |  |  | **Women** | | **Men** | | **Women** | | **Men** | |
|  | **Test result** | **Overall** | **no** | **yes** | **no** | **yes** | **no** | **yes** | **no** | **yes** |
|  |  |  |  |  |  |  |  |  |  |  |
|  |  | n (%) | n (%) | n (%) | n (%) | n (%) | n (%) | n (%) | n (%) | n (%) |
| **Five most frequent AABs:** |  |  |  |  |  |  |  |  |  |  |
| **Rheumatoid factor (RF)** | normal | 207 (64.1) | 101 (66.9) | 24 (50.0) | 59 (67.8) | 23 (62.2) | 96 (62.7) | 29 (63.0) | 74 (64.9) | 8 (80.0) |
|  | not normal | 116 (35.9) | 50 (33.1) | 24 (50.0) | 28 (32.2) | 14 (37.8) | 57 (37.3) | 17 (37.0) | 40 (35.1) | 2 (20.0) |
| **B2 Glykoprotein IgM** | normal | 307 (95) | 141 (93.4) | 47 (97.9) | 83 (95.4) | 36 (97.3) | 144 (94.1) | 44 (95.7) | 109 (95.6) | 10 (100) |
|  | not normal | 16 (5) | 10 (6.6) | 1 (2.1) | 4 (4.6) | 1 (2.7) | 9 (5.9) | 2 (4.3) | 4 (4.4) | 0 (0) |
| **Cardiolipin IgG** | normal | 317 (98.1) | 147 (97.4) | 46 (95.8) | 87 (100) | 37 (100) | 149 (97.4) | 44 (95.7) | 114 (100) | 10 (100) |
|  | not normal | 6 (1.9) | 4 (2.6) | 2 (4.2) | 0 (0) | 0 (0) | 4 (2.6) | 2 (4.3) | 0 (0) | 0 (0) |
| **Cardiolipin IgM** | normal | 318 (98.5) | 148 (98.0) | 47 (97.9) | 86 (98.9) | 37 (100) | 150 (98.0) | 45 (97.8) | 113 (99.1) | 10 (100) |
|  | not normal | 5 (1.5) | 3 (2.0) | 1 (2.1) | 1 (1.1) | 0 (0) | 3 (2.0) | 1 (2.2) | 1 (0.9) | 0 (0) |
| **Anti-dsDNA** | normal | 319 (98.8) | 149 (98.7) | 48 (100) | 85 (97.7) | 37 (100) | 151 (98.7) | 46 (100) | 112 (98.2) | 10 (100) |
|  | not normal | 4 (1.2) | 2 (1.3) | 0 (0) | 2 (2.3) | 0 (0) | 2 (1.3) | 0 (0) | 2 (1.8) | 0 (0) |
| **AAB screening tests:** |  |  |  |  |  |  |  |  |  |  |
| **ANA nuclear** | normal | 217 (67.2) | 99 (65.6) | 29 (60.4) | 61 (70.1) | 28 (75.7) | 95 (62.1) | 33 (71.7) | 81 (71.1) | 8 (80.0) |
|  | not normal | 106 (32.8) | 52 (34.4) | 19 (39.6) | 26 (29.9) | 9 (24.3) | 58 (37.9) | 13 (28.3) | 33 (28.9) | 2 (20.0) |
| **ANA cytoplasmic** | normal | 299 (92.6) | 138 (91.4) | 42 (87.5) | 82 (94.3) | 37 (100) | 139 (90.8) | 41 (89.1) | 109 (95.6) | 10 (100) |
|  | not normal | 24 (7.4) | 13 (8.6) | 6 (12.5) | 5 (5.7) | 0 (0) | 14 (9.2) | 5 (10.9) | 5 (4.4) | 0 (0) |
| **ANA mitotic.** | normal | 318 (98.5) | 149 (98.7) | 46 (95.8) | 86 (98.9) | 37 (100) | 151 (98.7) | 44 (95.7) | 113 (99.1) | 10 (100) |
|  | not normal | 5 (1.5) | 2 (1.3) | 2 (4.2) | 1 (1.1) | 0 (0) | 2 (1.3) | 2 (4.3) | 1 (0.9) | 0 (0) |
| **ANCA** | normal | 273 (84.5) | 126 (83.4) | 39 (81.3) | 78 (89.7) | 30 (81.1) | 125 (81.7) | 40 (87.0) | 99 (86.8) | 9 (90.0) |
|  | not normal | 50 (15.5) | 25 (16.6) | 9 (18.8) | 9 (10.3) | 7 (18.9) | 28 (18.3) | 6 (13.0) | 15 (13.2) | 1 (10.0) |
| **cANCA** | normal | 316 (97.8) | 147 (97.4) | 47 (97.9) | 86 (98.9) | 36 (97.3) | 149 (97.4) | 45 (97.8) | 113 (99.1) | 9 (90.0) |
|  | not normal | 7 (2.2) | 4 (2.6) | 1 (2.1) | 1 (1.1) | 1 (2.7) | 4 (2.6) | 1 (2.2) | 1 (0.9) | 1 (10.0) |
| **pANCA** | normal | 280 (86.7) | 130 (86.1) | 40 (83.3) | 79 (90.8) | 31 (83.8) | 129 (84.3) | 41 (89.1) | 100 (87.7) | 10 (100) |
|  | not normal | 43 (13.3) | 21 (13.9) | 8 (16.7) | 8 (9.2) | 6 (16.2) | 24 (15.7) | 5 (10.9) | 14 (12.3) | 0 (0) |
| **ENA-Screening** | normal | 318 (98.5) | 149 (98.7) | 46 (95.8) | 87 (100) | 36 (97.3) | 152 (99.3) | 43 (93.5) | 113 (99.1) | 10 (100) |
|  | not normal | 5 (1.5) | 2 (1.3) | 2 (4.2) | 0 (0) | 1 (2.7) | 1 (0.7) | 3 (6.5) | 1 (0.9) | 0 (0) |
| **At least one non-normal AAB test** | normal | 119 (36.8) | 60 (39.7) | 9 (18.8) | 35 (40.2) | 15 (40.5) | 55 (35.9) | 14 (30.4) | 45 (39.5) | 5 (50.0) |
|  | not normal | 204 (63.2) | 91 (60.3) | 39 (81.3) | 52 (59.8) | 22 (59.5) | 98 (64.1) | 32 (69.6) | 69 (60.5) | 5 (50.0) |

**Table S5: Odds ratio (OR) and 95% confidence interval (95% CI) for the association between the diagnosis of at least one allergic disease (yes/no, exposure) and serum autoantibodies (non-normal vs. normal; outcomes), stratified by sex**

|  |  | **Raw model*** | | | |  | **Extended model**** | | | |
| --- | --- | --- | --- | --- | --- | --- | --- | --- | --- | --- |
| **AABs** | **sex** | **OR** | **95% CI** | | **p-value** |  | **OR** | **95% CI** | | **p-value** |
| RF | f | 1.060 | 0.551 | 2.038 | 0.862 |  | 1.204 | 0.611 | 2.373 | 0.592 |
|  | m | 0.705 | 0.177 | 2.815 | 0.621 |  | 0.845 | 0.183 | 3.893 | 0.828 |
|  |  |  |  |  |  |  |  |  |  |  |
| ANA nuclear | f | 0.650 | 0.327 | 1.295 | 0.221 |  | 0.654 | 0.320 | 1.337 | 0.244 |
|  | m | 0.954 | 0.238 | 3.828 | 0.947 |  | 0.891 | 0.200 | 3.970 | 0.880 |
|  |  |  |  |  |  |  |  |  |  |  |
| ANCA | f | 0.673 | 0.273 | 1.660 | 0.390 |  | 0.704 | 0.275 | 1.803 | 0.465 |
|  | m | 0.665 | 0.079 | 5.622 | 0.708 |  | 0.385 | 0.036 | 4.068 | 0.427 |
|  |  |  |  |  |  |  |  |  |  |  |
| At least one non-normal AAB test | f | 1.427 | 0.716 | 2.840 | 0.312 |  | 1.294 | 0.614 | 2.727 | 0.499 |
|  | m | 0.796 | 0.229 | 2.766 | 0.719 |  | 0.561 | 0.137 | 2.290 | 0.420 |

* adjusted for age
** adjusted for age, BMI, sport (yes/no), CRP, EPA, ß-carotene

**Table S6: Comparison of association between allergic sensitization (yes/no; exposure) with different threshold (CAP-class 1 >= 0.35 kUA/l; CAP-class 2 >= 0.7 kUA/l) and outcomes serum autoantibodies (non-normal vs. normal; (high-)positive vs. non-positive), stratified by sex**

| **Outcome** | **Sex** | **Cut-off** | **Sensitized** (n) | **Total** (n) | **OR** | **95% CI** | | **P-value** |
| --- | --- | --- | --- | --- | --- | --- | --- | --- |
| At least one non-normal AAB test | female | CAP ≥ 0.70 | 48 | 199 | 3.17 | 1.367 | 7.342 | 0.007 |
| At least one non-normal AAB test | female | CAP ≥ 0.35 | 56 | 199 | 2.62 | 1.229 | 5.587 | 0.013 |
|  |  |  |  |  |  |  |  |  |
| At least one non-normal AAB test | male | CAP ≥ 0.70 | 37 | 124 | 1.01 | 0.427 | 2.401 | 0.978 |
| At least one non-normal AAB test | male | CAP ≥ 0.35 | 42 | 124 | 0.92 | 0.393 | 2.147 | 0.845 |
| At least one (high-)positive AAB test | female | CAP ≥ 0.70 | 48 | 199 | 1.99 | 0.996 | 3.978 | 0.051 |
| Aleast one (high-)positive AAB test | female | CAP ≥ 0.35 | 56 | 199 | 1.95 | 1.013 | 3.741 | 0.046 |
|  |  |  |  |  |  |  |  |  |
| At least one (high-)positive AAB test | male | CAP ≥ 0.70 | 37 | 124 | 0.9 | 0.361 | 2.263 | 0.828 |
| At least one (high-)positive AAB test | male | CAP ≥ 0.35 | 42 | 124 | 0.78 | 0.315 | 1.928 | 0.589 |

*All results for the extended models adjusted for age, BMI, sport (yes/no), CRP, EPA, ß-carotene*
